# Supplementary material for: Multivalency transforms SARS-CoV-2 antibodies into ultrapotent neutralizers
Source: Nat Commun. 2021 Jun 16;12:3661. doi: 10.1038/s41467-021-23825-2 (PMC8209050; doi:10.1038/s41467-021-23825-2)
Supplement: Supplementary file 1 — Supplementary Information [file 41467_2021_23825_MOESM1_ESM.pdf]

Supplementary Information

Multivalency transforms SARS-CoV-2 antibodies into ultrapotent neutralizers

Rujas *et al.*

Supplementary Figure 1

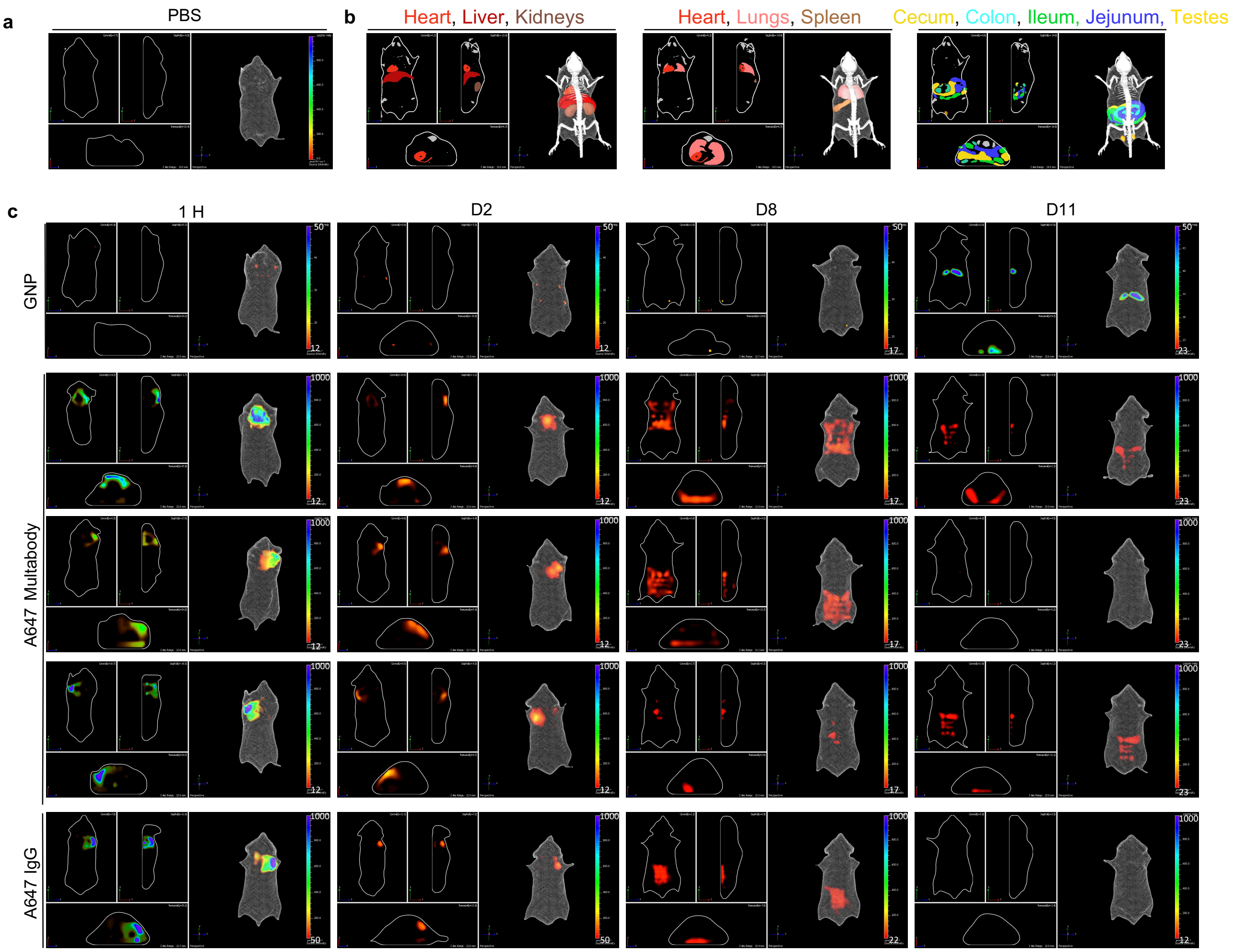

3D biodistribution of a surrogate mouse Multabody is comparable to its parental IgG. The biodistribution of 15 nm gold nanoparticles (GNP), MB and IgG samples labeled with Alexa-647 were visualized post subcutaneous injection into BALB/c mice via live non-invasive 3D whole body imaging. **a)** Representative 3D rendered fluorescent image overlaid with CT scan from PBS injected control. **b)** Depiction of the localization of major mouse organs overlaid with CT scan. **c)** 3D rendered fluorescent images overlaid with CT scan at 1 h (1H), two days (D2), eight days (D8) and 11 days (D11) post subcutaneous injection of gold nanoparticles (top), MB (three middle panels) or IgG (bottom panel). Each 3D image set is displayed showing dorsal view overlaid with CT scan (right), as well as a selected frontal (top left), medial (middle), and transverse (bottom left) planes based on signal localization. 3D fluorescent images were mapped to a rainbow look-up table (LUT), with color scale minimum set to background and maximum set to 50 pmol M^{-1} cm^{-1} (GNP) or 1000 pmol M^{-1} cm^{-1} (MB and IgG).

Supplementary Figure 2

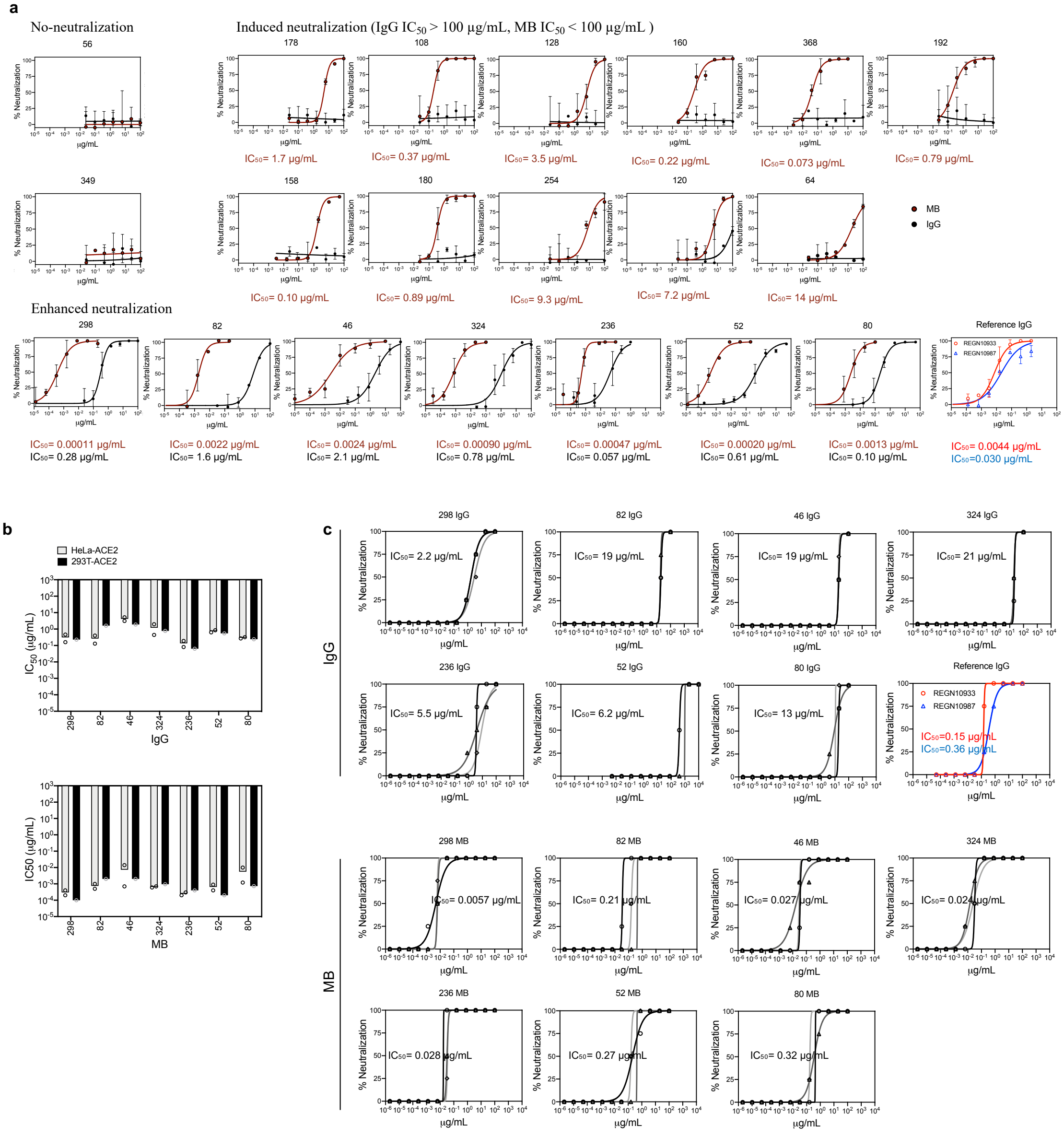

Neutralization of SARS-CoV-2 RBD-targeting Multabodies and their parental IgGs. **a**) Representative neutralization titration curves of 20 antibodies against SARS-CoV-2 PsV when displayed as IgGs (black) and MBs (dark red). The mean IC_{50} values of three biological replicates are displayed for comparison. The mean values ± SD for two technical replicates are shown in each neutralization plot. Source data are provided as a Source Data file. **b)** Neutralization profiles of selected IgGs and MBs against SARS-CoV-2 PsV targeting 293T-ACE2 (black) and HeLa-ACE2 (gray) target cells. The mean IC_{50} value and individual IC_{50} values of three and two biological replicates are shown for 293T-ACE2 and HeLa-ACE2 cells, respectively. **c)** Neutralization titration curves of three biological replicates (different shades of gray) against the authentic SARS-CoV-2/SB2-P4-PB strain. The mean IC_{50} is indicated. Neutralization potencies of recombinant mAbs REGN10933 (red) and REGN10987 (blue) are included in **(a)** and **(c)** as benchmarks for comparison.

Supplementary Figure 3

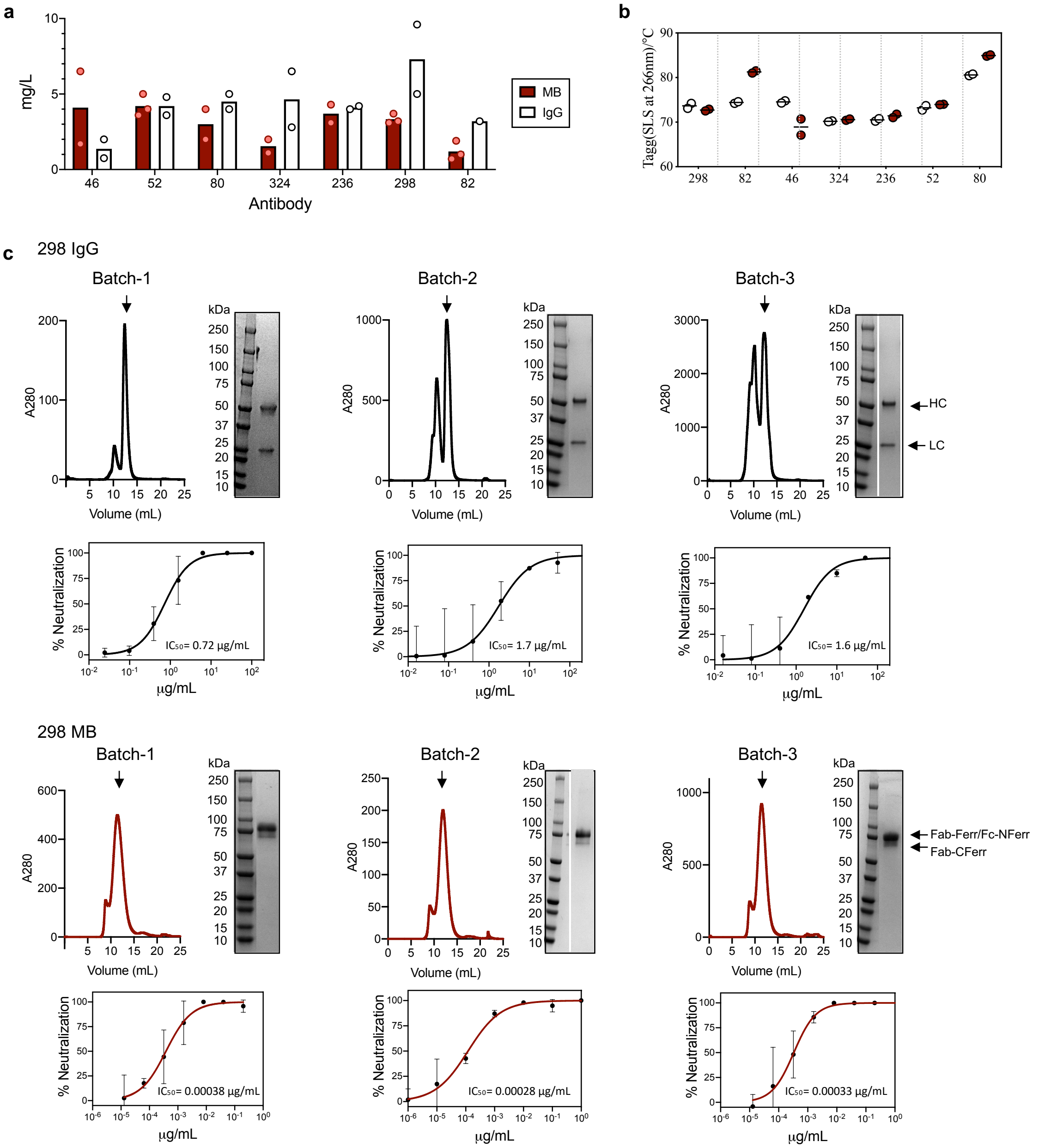

Expression yields and homogeneity of SARS-CoV-2 RBD-targeting Multabodies. **a)** Yield (mg/L) of the seven most potent IgGs (white) and their respective MBs (dark red). Mean values ± SD for two biologically independent samples. **b)** Aggregation temperature (T_{agg}, ºC) comparison as in **(a)**. The solid line denotes the mean T_{agg} value of two biologically independent samples. **c)** SEC chromatograms of 298 IgG (top row, black) and 298 MB (bottom row, dark red) from three independent expressions and purifications. Prior to SEC, in both cases, the samples were purified using Protein A affinity chromatography. The arrows indicate the peak used to perform a PsV neutralization assay from each batch. IC_{50} values (μg/mL) are noted. Mean values ± SD for two technical replicates are shown in each neutralization plot.

Supplementary Figure 4

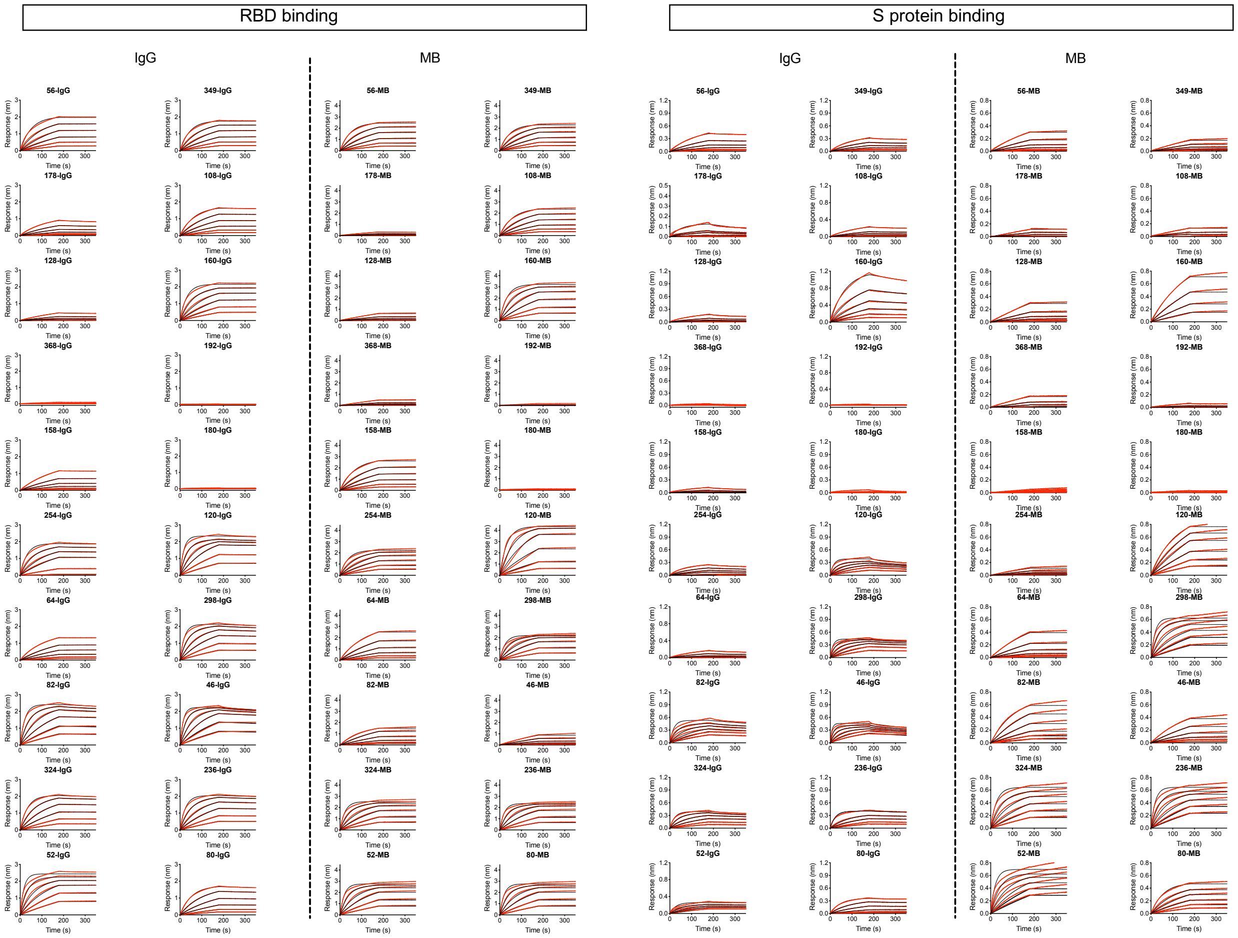

Binding profiles of IgGs and MBs. Sensograms of IgGs and MBs binding to RBD (left) and S protein (right) of SARS-CoV-2 immobilized onto Ni-NTA biosensors. 2-fold dilution series from 125 to 4 nM (IgG), and 16 to 0.5 nM (MB) were used. Red lines represent raw data, whereas black lines represent global fits.

Supplementary Figure 5

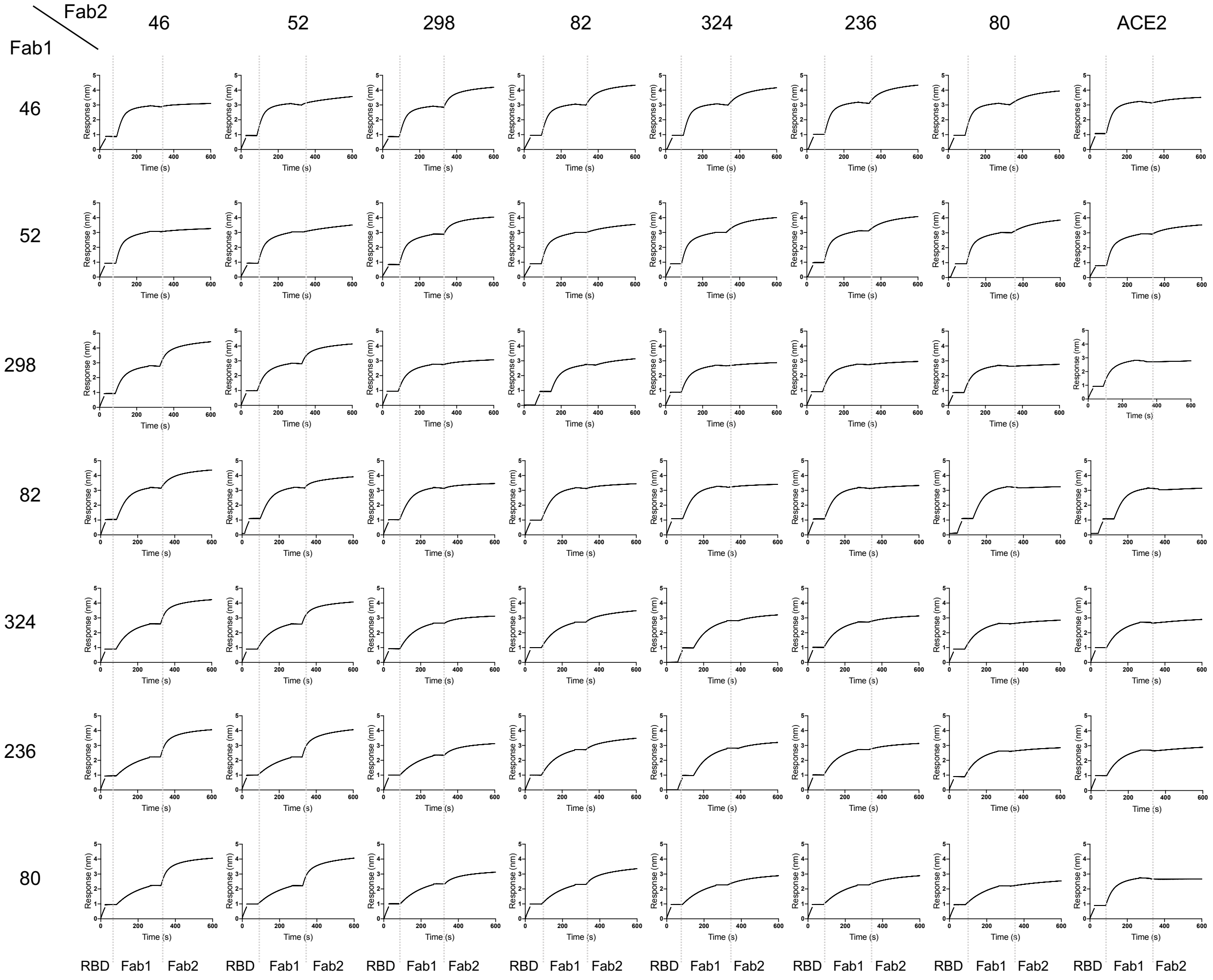

Epitope binning. mAb binding competition experiments to His-tagged RBD as measured by biolayer interferometry (BLI). 50 μg/ml of mAb 1 was incubated for 3 min followed by incubation with 50 μg/ml of mAb 2 for 5 min.

Supplementary Figure 6

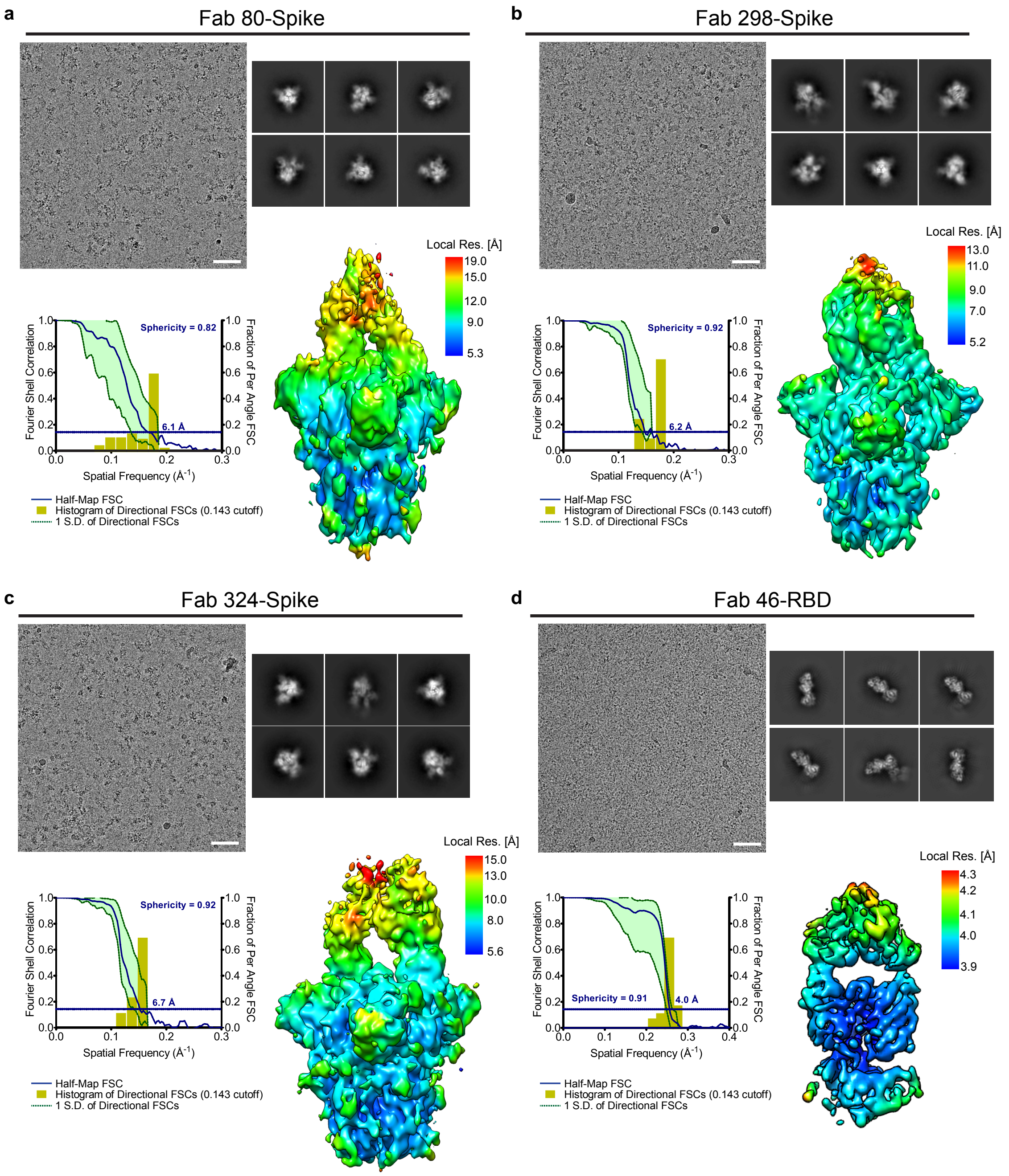

Cryo-EM analysis of the Fab-Spike and Fab-RBD complexes. Representative cryo-EM micrograph (scale bar 50 nm, top left), selected 2D class averages (top right), Fourier shell correlation curve from the final 3D non-uniform refinement (bottom left) and local resolution (Å) plotted on the surface of the cryo-EM map (bottom right) are shown for the Fab 80-Spike complex **(a)**, the Fab 298-Spike complex **(b)**, the Fab 324-Spike complex **(c)**, and the Fab 46-RBD complex **(d)**.

Supplementary Figure 7

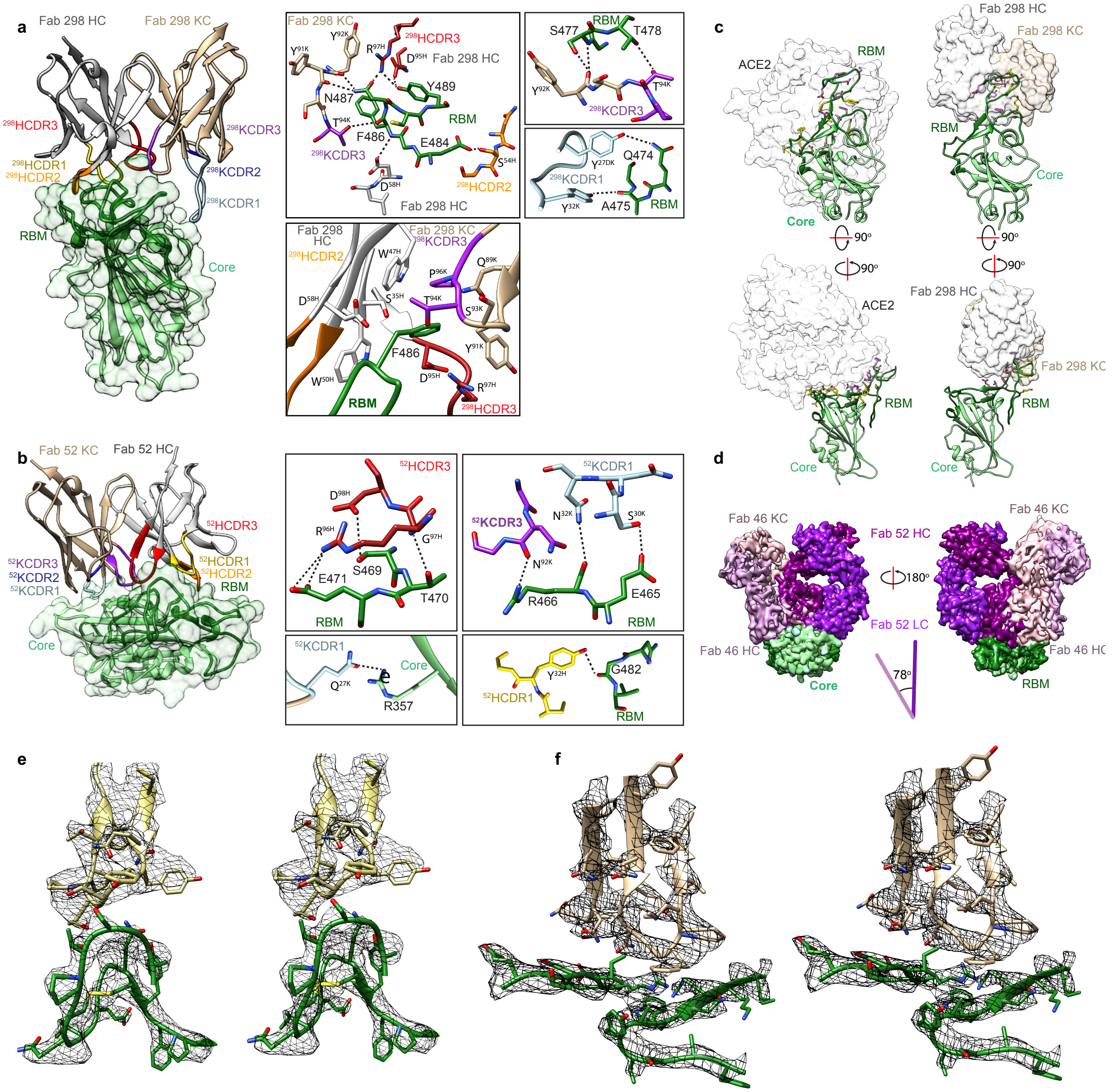

Binding interfaces of Fabs 52 and 298 and the RBD. Interaction of Fab 298 **(a)** and 52 **(b)** with RBD (light and dark green for the core and RBM regions, respectively) is mediated by complementarity determining regions (CDR) heavy (H) 1 (yellow), H2 (orange), H3 (red), kappa light (K) 1 (light blue) and K3 (purple). Critical binding residues are shown as sticks (insets). H-bonds and salt bridges are depicted as black dashed lines. L and H chains of Fabs are shown in tan and white, respectively. **c)** Bottom and side views of ACE2 (left) and Fab 298 (right) bound to RBD. RBD side-chains that are part of the binding interface of the ACE2-RBD and Fab 298-RBD complexes are depicted in pink, while RBD side-chains unique to a given interface are shown in yellow. Surfaces of ACE2, variable regions of Fab 298 HC and Fab 298 KC are shown in white, grey and tan, respectively. The RBD is colored as in **(a)**. **d)** Superposition of Fabs 46 (light pink) and 52 (dark pink) when bound to the RBD (green) reveals a distinct angle of approach for the two mAbs. Stereo-image of the composite omit map electron density contoured at 1.3 sigma at the interfaces of **e)** 298-RBD and **f)** 52-RBD.

Supplementary Figure 8

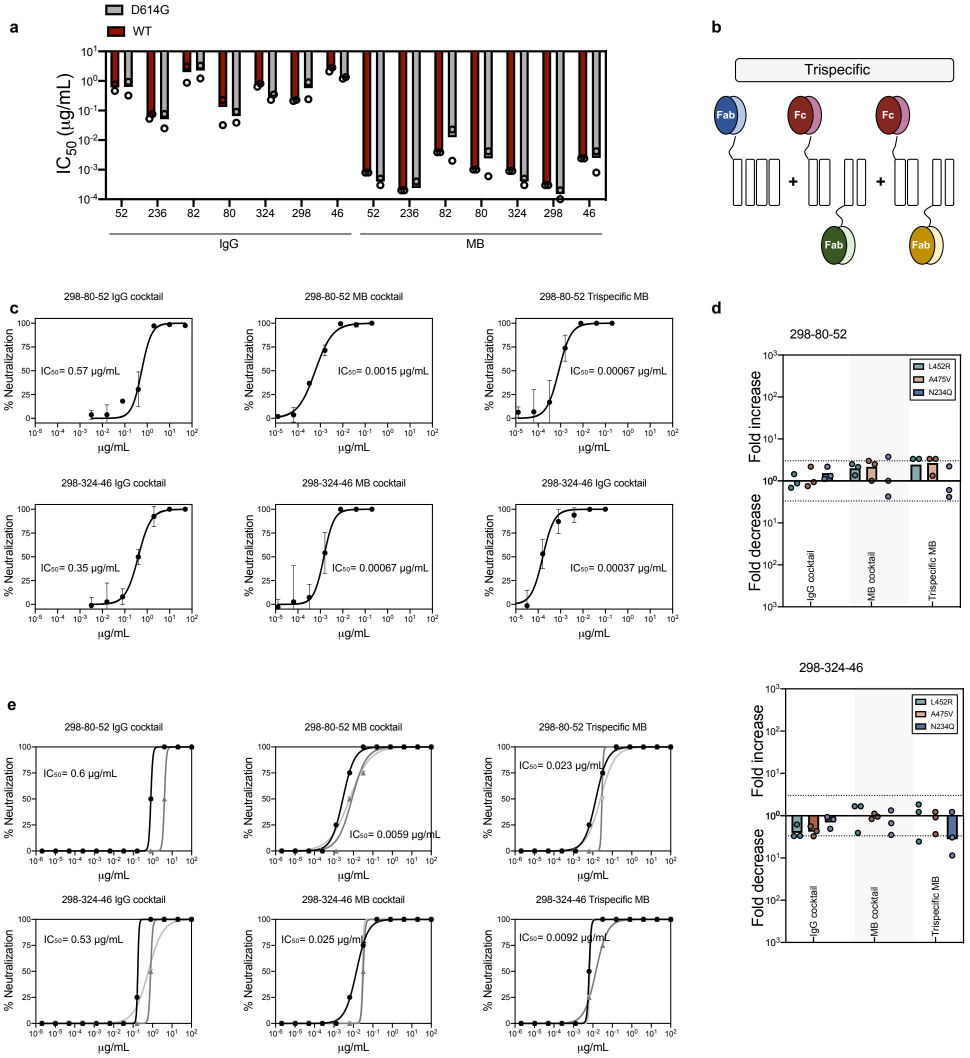

MBs potently overcome SARS-CoV-2 sequence variability. **a)** Comparison of the neutralization potency of selected IgGs and MBs against WT PsV (dark red) and the more infectious D614G PsV (grey). **b)** Schematic representation of a tri-specific MB generated by combination of three Fab specificities and the Fc fragment using the MB split design. **c)** Cocktails and tri-specific MBs that combine the specificities of mAbs 298, 80 and 52, or 298, 324 and 46 were generated and tested against WT PsV. The mean values ± SD for two technical replicates is represented in each representative neutralization plot. Source data are provided as a Source Data file. **d)** Neutralization potency change of cocktails and tri-specific MBs against pseudotyped SARS-CoV-2 variants in comparison to WT PsV. PsV variants that were sensitive to individual antibodies within the cocktails were selected. The area within the dotted lines represents a 3-fold change in IC_{50} value. This threshold was established as the cut-off for increased sensitivity (up bars) or increased resistance (down bars). **e)** Neutralization titration curves showing three biological replicates of cocktails and tri-specific MBs against the authentic SARS-CoV-2/SB2-P4-PB strain. Mean IC_{50} values of three biologically independent replicates are shown.

Supplementary Table 1. Cryo-EM data collection and image processing.

	Fab 80-Spike	Fab 298-Spike	Fab 324-Spike	Fab 46-RBD
EMDB ID	EMD-22739	EMD-22740	EMD-22741	EMD-22738
Data Collection				
Electron microscope	Titan Krios G3	Titan Krios G3	Titan Krios G3	Titan Krios G3
Camera	Falcon 4EC	Falcon 4EC	Falcon 4EC	Falcon 4EC
Voltage (kV)	300	300	300	300
Nominal magnification	75,000	75,000	75,000	75,000
Calibrated physical pixel size (Å)	1.03	1.03	1.03	1.03
Total exposure (e- /Å^2)	44	44	44	44
Number of frames	29	29	29	29
Image Processing				
Motion correction software	*cryoSPARC v2, Relion MotionCorr*	*cryoSPARC v2, Relion MotionCorr*	*cryoSPARC v2, Relion MotionCorr*	*cryoSPARCv2*
CTF estimation software	*cryoSPARCv2*	*cryoSPARCv2*	*cryoSPARCv2*	*cryoSPARCv2*
Particle selection software	*cryoSPARCv2*	*cryoSPARCv2*	*cryoSPARCv2*	*cryoSPARCv2*
3D map classification and refinement software	*cryoSPARCv2*	*cryoSPARCv2*	*cryoSPARCv2*	*cisTEM*
Micrographs used (total)	3610	7772	4478	4722
0˚ tilt	820	4259	1098	4722
40˚ tilt	2790	3513	3380	0
Global resolution (Å)	6.2	6.2	6	4
Particles in final maps	7,525	26,972	18,595	32,283

Supplementary Table 2. X-ray crystallography data collection and refinement statistics

	Fab 52- Fab 298-SARS-CoV-2 RBD
PDB ID	7K9Z
Data Collection	
Wavelength (Å)	1.03317
Space group	P 3_2 2 1
Cell dimensions	
a,b,c (Å)	87.6, 87.6, 325.1
α, β, γ (°)	90.0, 90.0, 120.0
Resolution (Å)	39.66-2.95 (3.05-2.95)
No. molecules in ASU	1
No. total observations	496,550 (43,958)
No unique observations	31,545 (3,060)
Multiplicity	15.7 (14.3)
R_{merge} (%)	16.8 (74.2)
R_{pim} (%)	4.3 (20.1)
$\langle I/\boldsymbol{\sigma} I\rangle$	12.3 (1.4)
$CC_{1/2}$	99.8 (86.3)
Completeness (%)	99.9 (99.9)
Refinement	
Non-hydrogen atoms	8061
Macromolecule	8047
Glycan	14
R_{work}/R_{free}	0.259/0.286
Rms deviations	
Bond lengths (Å)	0.002
Bond angles (°)	0.53
Ramachandran plot	
Favored regions (%)	95.6
Allowed regions (%)	4.1
Outliers (%)	0.3
Rotamer Outliers (%)	2.6
B-factors (Å^2)	
Wilson B-factor	78.6
Average B-factors	103.9
Average macromolecule	103.9
Average glycan	114.3

Supplementary Table 3. RBD-298 and RBD-52 contacting residues identified by PISA.

RBD	Residue	BSA (Å^2)	Interaction	Fab 298 (H-HC, K-KC)
453	Tyr	2	vdW	H-Thr31, H-Ile100
455	Leu	20	vdW	H-Thr31, H-Ile100
456	Phe	30	vdW	H-Thr31, H-Tyr32
458	Lys	1	vdW	K-Ser27F
474	Gln	12	vdW	K-Tyr27D
	Gln^{NE2}		HB	$K\text{-}Tyr27D^{OH}$
475	Ala	45	vdW	K-Tyr27D, H-Tyr32, K-Tyr32, H-Arg97
	Ala^{O}		HB	$K\text{-}Tyr32^{OH}$
476	Gly	23	vdW	K-Tyr27D, K-Tyr32, K-Tyr91, K-Tyr92, K-Ser93, H-Arg97
477	Ser	75	vdW	K-Tyr27D, K-Tyr92, K-Ser93, K-Thr94
	Ser^{N}		HB	$K\text{-}Tyr92^{O}$
	Ser^{OG}		HB	$K\text{-}Tyr92^{O}$
478	Thr	41	vdW	K-Tyr27D, K-Tyr92, K-Ser93, K-Thr94
	Thr^{OG1}		HB	$K\text{-}Thr94^{OG1}$
484	Glu	74	vdW	H-Trp50, H-Ser52, H-Ser54, H-Gly55, H-Gly56, H-Thr57, H-Asp58
	Glu^{OE2}		HB	$H\text{-}Ser54^{OG}$
485	Gly	28	vdW	H-Trp50, H-Thr57, H-Asp58
486	Phe	169	vdW	K-Gln89, K-Tyr91, K-Ser93, K-Thr94, K-Pro96, H-Ser35, H-Trp47, H-Trp50, H-Asp58, H-Asp95, H-Arg97
	Phe^{N}		HB	$H\text{-}Asp58^{OD2}$
	Phe^{O}		HB	$K\text{-}Thr94^{OG1}$
487	Asn	41	vdW	K-Tyr32, K-Tyr91, K-Ser93, K-Thr94, H-Asp95, H-Arg97
	Asn^{OD1}		HB	$H\text{-}Arg97^{NH2}$
	Asn^{ND2}		HB	$K\text{-}Tyr91^{O}$, $K\text{-}Tyr92^{O}$
488	Cys	1	vdW	H-Trp50

489	Tyr	84	vdW	H-Ser30, H-Thr31, H-Tyr32, H-Trp50, H-Asp95, H-Arg97,
	Tyr^{OH}		HB	H-Arg97^{NH2}, H-Asp95^{OD2}
493	Gln	45	vdW	H-Ser30, H-Thr31, H-Ile100
Total BSA ($Å^2$):		691		

RBD	Residue	BSA ($Å^2$)	Interaction	Fab 52 (H-HC, K-KC)
346	Arg	45	vdW	H-Gln64
351	Tyr	29	vdW	K-Phe94, H-Ile52, H-Thr56, H-Asn58
352	Ala	19	vdW	K-Gly93, K-Phe94
354	Asn	6	vdW	K-Phe94
355	Arg	36	vdW	K-Ser0, K-Gln27
	Arg^{O}		HB	K-Ser0^{OG}
356	Lys	13	vdW	K-Ser0
357	Arg	66	vdW	K-Ser0, K-Gln27
	Arg^{N}		HB	K-Ser0^{OG}
	Arg^{NH2}		HB	K-Gln27^{OE1}
449	Tyr	13	vdW	H-Phe554, H-Thr56
450	Asn	38	vdW	H-Phe554, H-Thr56
452	Leu	47	vdW	H-Ile52, H-Phe554, H-Gly55, H-Thr56
462	Lys	1	vdW	K-Ser30
465	Glu	28	vdW	K-Ser30, K-Asn31, K-Asn32, K-Asn92
	Glu^{OE2}		HB	K-Ser30^{OG}
466	Arg	70	vdW	K-Asn32, K-Asn92, K-93Gly, K-Phe94
	Arg^{O}		HB	K-Asn32^{ND2}
	Arg^{NH1}		HB	K-Asn92^{O}
467	Asp	12	vdW	K-Asn32, K-Asn92, H-Asp98
468	Ile	102	vdW	K-Asn32, K-Gly91, K-Asn92, K-93Gly, K-Phe94, K-Leu96, H-Arg96, H-Gly97, H-Asp98
469	Ser	37	vdW	K-Asn32, H-Arg96, H-Gly97, H-Asp98
	Ser^{OG}		HB	H-Asp98^{OD1}
470	Thr	67	vdW	H-Ser31, H-Tyr32, H-Gly33, H-Ile52, H-Asp95, H-Arg96, H-Gly97, H-Asp98

	Thr^{OG1}		HB	H-$Gly97^{N}$
471	Glu	39	vdW	H-Tyr32, H-Arg96, H-Gly97, H-Asp98
	Glu^{OE1}		SB	H-$Arg96^{NH2}$
	Glu^{OE2}		SB	H-$Arg96^{NH2}$
472	Ile	7	vdW	H-Ser31, H-Tyr32, H-Arg96
481	Asn	1	vdW	H-Tyr32
482	Gly	42	vdW	H-Ser31, H-Tyr32
	Gly^{O}		HB	H-$Tyr32^{OH}$
483	Val	12	vdW	H-Thr28, H-Ser31, H-Tyr32
484	Glu	53	vdW	H-Thr28, H-Phe29, H-Thr30, H-Ser31, H-Met54
490	Phe	87	vdW	H-Thr30, H-Ser31, H-Tyr32, H-Gly33, H-Ile52, H-Met54, H-Phe554
492	Leu	13	vdW	H-Ile52, H-Met54, H-Phe554
493	Gln	8	vdW	H-Phe554
494	Ser	17	vdW	H-Phe554
Total BSA ($Å^2$):		904		

Fab 298	Residue-Chain	BSA ($Å^2$)	Interaction	RBD
30	Ser-H	9	vdW	Tyr489, Gln493
31	Thr-H	68	vdW	Tyr453, Leu455, Phe456, Tyr489, Gln493
32	Tyr-H	57	vdW	Phe456, Ala475, Tyr489
35	Ser-H	8	vdW	Phe486
47	Trp-H	23	vdW	Phe486
50	Trp-H	79	vdW	Glu484, Gly485, Phe486, Cys488, Tyr489
52	Ser-H	8	vdW	Glu484
54	Ser-H	17	vdW	Glu484
	Ser^{OG}-H		HB	$Glu484^{OE2}$
55	Gly-H	7	vdW	Glu484
56	Gly-H	7	vdW	Glu484
57	Thr-H	10	vdW	Glu484, Gly485
58	Asp-H	28	vdW	Glu484, Gly485, Phe486

	Asp^{OD2}-H		HB	Phe486^{N}
95	Asp-H	15	vdW	Phe486, Asn487, Tyr489
	Asp^{OD2}-H		HB	Tyr489^{OH}
97	Arg-H	61	vdW	Asn487, Tyr489
	Arg^{NH2}-H		HB	Asn487^{OD1}, Tyr489^{OH}
100	Ile-H	7	vdW	Tyr453, Leu455, Gln493
27D	Tyr-K	59	vdW	Gln474, Ala475, Gly476, Ser477, Thr478
	Tyr^{OH}-K		HB	Gln474^{NE2}
27F	Ser-K	1	vdW	Lys458
32	Tyr-K	28	vdW	Ala475, Gly476, Asn487
	Tyr^{OH}-K		HB	Ala475^{O}
89	Gln-K	1	vdW	Phe486
91	Tyr-K	30	vdW	Gly476, Phe486, Asn487
	Tyr^{O}-K		HB	Asn487^{ND2}
92	Tyr-K	40	vdW	Gly476, Ser477, Thr478
	Tyr^{O}-K		HB	Asn487^{ND2}
	Tyr^{O}-K		HB	Ser477^{N}, Ser477^{OG}
93	Ser-K	26	vdW	Gly476, Ser477, Thr478, Phe486, Ser487
94	Thr-K	57	vdW	Ser477, Thr478, Phe486, Asn487
	Thr^{OG1}-K		HB	Thr478^{OG1}, Phe486^{O}
96	Pro-K	23	vdW	Phe486
Total BSA (Å^2):			669	

Fab 52	Residue-Chain	BSA (Å^2)	Interaction	RBD
28	Thr-H	24	vdW	Val483, Glu484
30	Thr-H	3	vdW	Glu484, Phe490
31	Ser-H	66	vdW	Glu471, Gly482, Val483, Glu484, Phe490
32	Tyr-H	51	vdW	Thr470, Glu471, Ile472, Asn481, Gly482, Val483, Phe490
	Tyr^{OH}-H		HB	Gly482^{O}

33	Gly-H	7	vdW	Thr470, Phe490
52	Ile-H	62	vdW	Tyr351, Leu452, Thr470, Phe490, Leu492
53	Met-H	38	vdW	Glu484, Phe490, Leu492
54	Phe-H	110	vdW	Tyr449, Asn450, Leu452, Phe490, Leu492, Gln493, Ser494
55	Gly-H	4	vdW	Leu452
56	Thr-H	57	vdW	Tyr351, Tyr449, Asn450, Leu452
58	Asn-H	10	vdW	Tyr351
64	Gln-H	43	vdW	Arg346
95	Asp-H	2	vdW	Thr470
96	Arg-H	64	vdW	Ile468, Ser469, Thr470, Glu471, Ile472
	Arg^{NH2}-H		SB	$Glu471^{OE1}$, $Glu471^{OE2}$
97	Gly-H	64	vdW	Ile468, Ser469, Thr470, Glu471
	Gly^{N}-H		HB	$Thr470^{OG1}$
98	Asp-H	28	vdW	Asp467, Ile468, Ser469, Thr470, Glu471
	Asp^{OD1}-H		HB	$Ser469^{OG}$
0	Ser-K	77	vdW	Arg355, Lys356, Arg357
	Ser^{OG}-K		HB	$Arg355^{O}$, $Arg357^{N}$
27	Gln-K	60	vdW	Arg355, Arg357
	Gln^{OE1}-K		HB	$Arg357^{NH2}$
30	Ser-K	28	vdW	Lys462, Glu465
	Ser^{OG} -K		HB	$Glu465^{OE2}$
32	Asn-K	19	vdW	Glu465, Arg466, Asp467, Ile468, Ser469
	Asn^{ND2}-K		HB	$Arg466^{O}$
91	Gly-K	18	vdW	Ile468
92	Asn-K	56	vdW	Glu465, Arg466, Asp467, Ile468
	Asn^{O}-K		HB	$Arg466^{NH1}$
93	Gly-K	22	vdW	Ala352, Arg466, Ile468
94	Phe-K	56	vdW	Tyr351, Ala352, Asn354, Arg466, Ile468
96	Leu-K	5	vdW	Ile468
Total BSA ($Å^2$):			974	

vdW: van der Waals interaction (5.0 Å cut-off)
HB: hydrogen bond (3.8 Å cut-off)
SB: salt bridge (4.0 Å cut-off)

Supplementary Table 4. Primer sequences

Primer name	Primer
N234Q_fwd	CAGATCACCCGGTTTCAGACACTGCTGGCC
N234Q_rev	GATGCCGATGGGCAGATCCACCAGGGG
L452R_fwd	CGGTACCGGCTGTTCCGGAAGTCCAATCTG
L452_rev	GTAATTGTAGTTGCCGCCGACTTTGG
A475V_fwd	GTGGGCAGCACCCCTTGTAACGGCGTGGAAG
A475V_rev	CTGATAGATCTCGGTGGAGATGTCCC
V483A_fwd	GCCGAAGGCTTCAACTGCTACTTCCCACTGC
V483A_rev	GCCGTTACAAGGGGTGCTGCCGGCC
N439K_fwd	AAGAACCTGGACTCCAAAGTCGGCGGCAACTAC
N439K_rev	GCTGTTCCAGGCAATCACACAGCCGGTG

Supplementary Data 1. Sequence, biophysical and functional details of antibodies.
