# Supplementary material for: Multivalency transforms SARS-CoV-2 antibodies into ultrapotent neutralizers
Source: Nat Commun. 2021 Jun 16;12:3661. doi: 10.1038/s41467-021-23825-2 (PMC8209050; doi:10.1038/s41467-021-23825-2)
Supplement: Supplementary file 3 — Description of Additional Supplementary Files [file 41467_2021_23825_MOESM3_ESM.pdf]

Description of Additional Supplementary Files

File Name: Supplementary Data 1
Description: Information of the monoclonal antibody sequences, their binding affinities to RBD and neutralization IC50 values.
