# Supplementary material for: Multivalency transforms SARS-CoV-2 antibodies into ultrapotent neutralizers
Source: Nat Commun. 2021 Jun 16;12:3661. doi: 10.1038/s41467-021-23825-2 (PMC8209050; doi:10.1038/s41467-021-23825-2)
Supplement: Supplementary file 5 — Reporting Summary [file 41467_2021_23825_MOESM5_ESM.pdf]

nature research

Corresponding author(s): Jean-Philippe Julien

Last updated by author(s): May 6, 2021

Reporting Summary

Nature Research wishes to improve the reproducibility of the work that we publish. This form provides structure for consistency and transparency in reporting. For further information on Nature Research policies, see our Editorial Policies and the Editorial Policy Checklist.

Statistics

For all statistical analyses, confirm that the following items are present in the figure legend, table legend, main text, or Methods section.

n/a	Confirmed	
☐	☒	The exact sample size (*n*) for each experimental group/condition, given as a discrete number and unit of measurement
☐	☒	A statement on whether measurements were taken from distinct samples or whether the same sample was measured repeatedly
☒	☐	The statistical test(s) used AND whether they are one- or two-sided<br>*Only common tests should be described solely by name; describe more complex techniques in the Methods section.*
☒	☐	A description of all covariates tested
☒	☐	A description of any assumptions or corrections, such as tests of normality and adjustment for multiple comparisons
☐	☒	A full description of the statistical parameters including central tendency (e.g. means) or other basic estimates (e.g. regression coefficient) AND variation (e.g. standard deviation) or associated estimates of uncertainty (e.g. confidence intervals)
☒	☐	For null hypothesis testing, the test statistic (e.g. *F*, *t*, *r*) with confidence intervals, effect sizes, degrees of freedom and *P* value noted<br>*Give P values as exact values whenever suitable.*
☒	☐	For Bayesian analysis, information on the choice of priors and Markov chain Monte Carlo settings
☒	☐	For hierarchical and complex designs, identification of the appropriate level for tests and full reporting of outcomes
☒	☐	Estimates of effect sizes (e.g. Cohen's *d*, Pearson's *r*), indicating how they were calculated

Our web collection on statistics for biologists contains articles on many of the points above.

Software and code

Policy information about availability of computer code

Data collection	X-ray diffraction data were collected at the 23-ID-D beamline at the Argonne National Laboratory Advanced Photon Source (APS) at the Argonne National Laboratory.<br>Cryo-EM data was acquired at 300 kV with a Thermo Fisher Scientific Titan Krios G3 electron microscope and prototype Falcon 4 camera operating in electron counting mode at 250 frames/s. Data collection was monitored with cryoSPARC Live.<br>Octet data acquisition by BLI was done using ForteBio Data Acquisition software version 9.0.0.13.<br>Stability experiments were carried out using Uni Client 2.2.0.0 and data was analyzed with the Unit Analysis software v2.2.1.<br>Dynamic light scattering (DLS) experiments were performed and analyzed using the DYNAMICS software version 7.10.1.21
Data analysis	X-ray crystallography data of the 52-298-RBD ternary complex was processed and scaled using XDS. The structure were determined by molecular replacement using Phaser (version 1.17.1_3660). CNTO88 Fab was used as a model for 52 Fab (PDB ID: 4DN3), 20358 Fab was used as a model for 298 Fab (PDB ID: 5CZX), and PDB ID: 6XDG was used as a search model for the RBD. Refinement of the structure was carried out by iterative rounds of phenix.refine (version 1.17.1_3660) and manual building in Coot (version 0.8). SBGrid was used to access all crystallography software. Pymol (Version 2.1.1) was used to generate the models for the figures.<br>CTF estimation, particle selection, 3D map classification and refinement of the cryo-EM data for Fab 80-Spike, Fab 298-Spike and Fab 324-Spike has been done using CryoSPARC. In addition, CryoSPARC patch motion and Relion MotionCorr was used to correct for preferred orientation. In the case of Fab 46-RBD complex, CryoSPARC was used to perform motion correction, CTF estimation and particle selection while cisTEM was used for 3D map classification and refinement.<br>Octet data analysis was performed using version 9.0.0.6 of ForteBio Data Analysis software.<br>DYNAMICS software version 7.10.1.21 was used to analyze thermostability data obtain by Dynamic light scattering (DLS)<br>Biodistribution data was analyzed using the IVIS software (Living Image Software for IVIS version 4.7.3).<br>Prism (version 8.2.0) was used to plot affinity values from BLI, SARS-CoV-2 pseudovirus and authentic virus neutralization.

SPR data analysis was performed usingCarterra's Kinetic Inspection Tool (version Oct 2019)

For manuscripts utilizing custom algorithms or software that are central to the research but not yet described in published literature, software must be made available to editors and reviewers. We strongly encourage code deposition in a community repository (e.g. GitHub). See the Nature Research guidelines for submitting code & software for further information.

Data

Policy information about availability of data

All manuscripts must include a data availability statement. This statement should provide the following information, where applicable:

- Accession codes, unique identifiers, or web links for publicly available datasets
- A list of figures that have associated raw data
- A description of any restrictions on data availability

The electron microscopy maps have been deposited at the Electron Microscopy Data Band (EMDB) with accession codes EMD-22738, EMD-22739, EMD-22740, EMD-22741 (Table S2- DOI:10.1101/2020.10.15.341636)). The crystal structure of 298-52-RBD complex (Table S3- DOI: 10.2210/pdb7K9Z/pdb) is available from the Protein Data Bank under accession code PDB: 7K9Z. Sequences of the monoclonal antibodies are available in Table S1. Other data are available from the corresponding author upon reasonable request.

Additional PDB entries were used throughout the manuscript to perform a comparative analysis of the different bins targeted by mAbs. The entries used in that analysis are: REGN10933 (PDB ID:6XDG), CV30 (PDB ID:6XE1), C105 (PDB ID:6XCM), COVA2-04 (PDB ID:7JMO), COVA2-39 (PDB ID:7JMP), CC12.1 (PDB ID:6XC2), BD23 (PDB ID:7BYR), B38 (PDB ID:7BZ5), P2C-1F11 (PDB ID:7BWJ), 2-4 (PDB ID:6XEY), CB6 (PDB ID:7C01), REGN10987 (PDB ID:6XDG), S309 (PDB ID:6WPS,6WPT), EY6A (PDB ID:6ZCZ), CR3022 (PDB ID:6YLA), H014 (PDB ID:7CAH), 4-8 (EMD ID:22159), 4A8 (PDB ID:7C2L) and 2-43 (EMDB ID:22275).

Field-specific reporting

Please select the one below that is the best fit for your research. If you are not sure, read the appropriate sections before making your selection.

[x] Life sciences [ ] Behavioural & social sciences [ ] Ecological, evolutionary & environmental sciences

For a reference copy of the document with all sections, see nature.com/documents/nr-reporting-summary-flat.pdf

Life sciences study design

All studies must disclose on these points even when the disclosure is negative.

Sample size	For binding kinetics: two replicates were performed. Since both replicate values differ between each other less than 2 fold we were confident with the value and hence we believed that we did not require to increase the number of replicates further. For neutralization experiments with WT PsV, PsV variants and authentic SARS-CoV-2 virus at least three individual experiments (biological replicates) have been performed. In case that a fold difference in the IC50 value bigger than 5-fold was obtained between replicates, an additional replicate was performed. In general the obtained IC50 values from three individual experiments met that criteria and hence we consider that sample size accurate enough to report mAb/MB pontecy.
Data exclusions	No data were excluded from the analyses.
Replication	In general attempts of replication were successful and normally we have performed three biological replicates. If a replicate did not meet our replication quality criteria (explained in Sample size), an additional replicate was performed to confirm.
Randomization	Due to the in vitro nature of our study, no randomization was necessary for our biophysical and structural studies
Blinding	Blinding was done in the case of neutralization experiments with authentic virus and in the case of epitope binning experiments. Cryo-EM structures were determined without knowledge of the epitope binning data. Blinding of some of the experiments were not possible since the person producing the biological samples was also performing some of the functional assay (PsV neutralization data, Fc-modified MB in vitro characterization and thermostability studies). In the case some of the binding experiments (to Spike variants) blinding was not necessary since that data was used to validate the crystallization data and not to generate new claims. Sera samples for PK analysis and ADA determination were blinded.

Reporting for specific materials, systems and methods

We require information from authors about some types of materials, experimental systems and methods used in many studies. Here, indicate whether each material, system or method listed is relevant to your study. If you are not sure if a list item applies to your research, read the appropriate section before selecting a response.

Materials & experimental systems

n/a	Involved in the study	
☐	☒	Antibodies
☐	☒	Eukaryotic cell lines
☒	☐	Palaeontology and archaeology
☐	☒	Animals and other organisms
☒	☐	Human research participants
☒	☐	Clinical data
☒	☐	Dual use research of concern

Methods

n/a	Involved in the study	
☒	☐	ChIP-seq
☒	☐	Flow cytometry
☒	☐	MRI-based neuroimaging

Antibodies

Antibodies used

The 20 IgG discovered from phage display whose sequences are in Table S1. In addition, several antibodies, which sequences have been obtained from the literature (4A8, BD23, CC12.1, CC6.30, CoVA 2-15, R10987, R10933, COV2-2196, COV2-2130). All antibodies were gene synthesized at GeneArt and produced recombinantly in our laboratory.

Validation

Binding of each of the antibodies used (those discovered by phage display and those obtained directly from the literature) have been tested against the SARS-CoV-2 Spike antigen by biolayer interferometry. In addition, their reported capacity to neutralize SARS-CoV-2 was confirmed with our lentiviral-based PsV neutralization assays.

Eukaryotic cell lines

Policy information about cell lines

Cell line source(s)

FreeStyle™ 293-F Cells (ThermoFisher R79007), HEK 293T (ATCC CRL-3216), VeroE6 (ATCC CRL-1586) and HeLa cells (kidly provided by D.R. Burton (The Scripps Research Institute)

Authentication

Cell lines were not authenticated.

Mycoplasma contamination

Cell lines were not tested for mycoplasma contamination.

Commonly misidentified lines
(See ICLAC register)

No commonly misidentified cell lines were used in the study.

Animals and other organisms

Policy information about studies involving animals; ARRIVE guidelines recommended for reporting animal research

Laboratory animals

8-week-old male BALB/c mice and 12-week-old male C57BL/6 mice were used in the study

Wild animals

The study did not involve wild animals

Field-collected samples

The study did not involve Field-collected samples

Ethics oversight

All procedures related to C57BL/6 mice were approved by the Local Animal Care Committee at the University of Toronto Scarborough.
All procedures regarding BALB/c mice were approved by the Local Animal Care Committee at the University of Toronto

Note that full information on the approval of the study protocol must also be provided in the manuscript.
